# Mitochondrial DNA haplogroup distribution in Chaoshanese with and without myopia

**Published:** 2010-02-26

**Authors:** Qin Wang, Panfeng Wang, Shiqiang Li, Xueshan Xiao, Xiaoyun Jia, Xiangming Guo, Qing-Peng Kong, Yong-Gang Yao, Qingjiong Zhang

**Affiliations:** 1 State Key Laboratory of Ophthalmology, Zhongshan Ophthalmic Center, Sun Yat-sen University, Guangzhou, China; 2 State Key Laboratory of Genetic Resources and Evolution, Kunming Institute of Zoology, Chinese Academy of Sciences, Kunming, China; 3 Key Laboratory of Animal Models and Human Disease Mechanisms of Chinese Academy of Sciences & Yunnan Province, Kunming Institute of Zoology, Kunming, China

## Abstract

**Purpose:**

Mitochondrial DNA (mtDNA) haplogroups affect the clinical expression of Leber hereditary optic neuropathy, age-related macular degeneration, and other diseases. The objective of this study is to investigate whether an mtDNA background is associated with myopia.

**Methods:**

Blood DNA was obtained from 192 college students, including 96 individuals with moderate-to-high myopia and 96 controls without myopia. All the subjects were from a well-known isolated population living in the Chaoshan area of east Guangdong Province and speaking one of the four major dialects in southern China. The mtDNA haplogroups in the 192 subjects were determined by sequencing the mtDNA control region and partial coding regions as well as by analysis of restriction fragment length polymorphisms. Each mtDNA was classified according to the updated version of the Eastern Asian haplogroup system.

**Results:**

Sixteen mtDNA haplogroups were recognized in the 192 subjects. The overall matrilineal structures of the samples with and without myopia were similar and had genetic imprints showing their ethno-origin. There was no statistical difference in frequencies of haplogroup distribution between subjects with and without myopia (χ^2^ test, p=0.556).

**Conclusions:**

We failed to identify clues that suggest an involvement of mtDNA background in the predisposition to myopia.

## Introduction

Mitochondrial bioenergetics is linked to oxidative stress that is associated with aging and neurodegeneration [1-3]. Mitochondria are involved in the production and clearance of reactive oxygen species (ROS), and mutations of mitochondrial DNA (mtDNA) may result in energy deficiency and an increase in oxygen radicals. mtDNA haplogroups, which are determined by a series of characteristic variations and were formed during the origin and migration of modern humans, have been shown to play active roles in several neurodegenerative diseases, including Alzheimer disease [4,5], Parkinson disease [6], and multiple sclerosis [7], despite some of the original claims not being repeated in subsequent studies [8]. In the eye, mtDNA haplogroups have been reported to affect the clinical expression of Leber hereditary optic neuropathy (LHON) in European [9] and Chinese families [10], age-related macular degeneration [11,12], and optic neuritis [13]. The mtDNA haplogroup effect is ethnic specific, as demonstrated in LHON where the haplogroups associated with LHON expression in Chinese populations are different from those in Caucasian populations [10].

Myopia can be caused by excessive reading and close work, which is potentially related to oxidative stress [14-16]. Individuals exposed to hyperbaric oxygen showed a refractive change to myopia [17-19]. On the other hand, high myopia is frequently associated with retinal neurodegeneration [20,21]. Under a similar environment and with similar reading behavior, some individuals develop myopia but others do not, suggesting a genetic background involvement. Linkage and association studies on the nuclear genome have demonstrated the importance of genetic factors in the development of myopia, especially high-grade myopia [22-25]. However, the exact molecular basis for most myopia remains unknown. There have been no reports on the potential association of myopia with the mitochondrial genome, although mtDNA variations and haplogroups are known to be associated with neurodegeneration and oxidative stress.

Chaoshanese is an intriguing, isolated, Han Chinese population that is located in the Chaoshan area, east Guangdong Province. This population has unique features in dialects, life styles, customs, habits, and a population census of 12 million. The Chaoshanese are suggested to be descendents of northern Chinese who immigrated during the Ming Dynasty (1368–1628 A.D.) or earlier [26]. In this study, we analyzed the mtDNA haplogroup distribution frequencies in Chaoshanese with and without myopia to detect the potential association between the mtDNA background and myopia.

## Methods

### Subjects

College students were recruited from 12 universities in Guangzhou, China, as part of a project to identify the genetic causes of complex high myopia. In total 2,699 students were examined, including 1,276 individuals with moderate-to-high myopia (spherical refraction at each meridian ≤–4.00D) and 1,423 control individuals without a significant refractive error (with best unaided visual acuity of 1.0 or better and bilateral refraction of a spherical equivalent between −0.50D and +2.00D). For this study, 96 cases (66 males and 33 females, age from 19 to 25) and 96 controls (66 males and 33 females, age from 19 to 26) from the Chaoshan area were selected based on similarities in age, gender, educational background, and ethnic origin (local dialect and places where they grew up). Detailed clinical information on the subjects is listed in Table 1. The 96 cases were selected based on the following criteria: 1) born in the Chaoshan area and can speak the Chaoshanese dialect; 2) best corrected visual acuity of 0.8 or better; 3) spherical refraction at each meridian ≤–4.00D; 4) no other known eye or related systemic diseases; 5) no family history of high myopia; and 6) myopia occurred at age 7 years or older). The 96 controls met the following criteria: 1) born in the Chaoshan area and can speak the Chaoshanese dialect; 2) best unaided visual acuity of 1.0 or better; 3) bilateral refraction between −0.50D and +2.00D (spherical equivalent); 4) no other known eye or related systemic diseases; and 5) no family history of high myopia or hyperopia. Case and control individuals without complete data, especially data for the measurement of IOL Master V5 (Carl Zeiss Meditec AG, Jena, Germany), were excluded.

**Table 1:** Information of the subjects with and without myopia.

**Characteristics**	**Myopias (M group; n=96)**		**Controls (NC group; n=96)**	
	OD	OS	OD	OS
Age, mean(SD), y	21.8 (1.3)		21.7 (1.3)	
Females, No. (%)	33 (34.4)		33 (34.4)	
SE, mean (SD), D	−6.52 (1.31)	−6.37 (1.36)	0.27 (0.51)	0.33 (0.44)
AL, mean(SD), mm	26.28 (0.96)	25.22 (1.02)	23.78 (0.72)	23.72 (0.68)
K, mean (SD), D	43.72 (1.42)	43.69 (1.43)	42.75 (1.45)	42.75 (1.46)
ACD, mean (SD), mm	3.78 (0.29)	3.79 (0.31)	3.44 (0.23)	3.47 (0.23)
Partial correlation with SE, r (p value)				
AL	−0.68 (<0.001)	−0.71 (<0.001)	−0.30 (0.003)	−0.34 (0.001)
K	−0.55 (<0.001)	−0.57 (<0.001)	−0.23 (0.025)	−0.21 (0.046)
ACD	0.28 (0.007)	0.19 (0.062)	0.01 (0.939)	0.08 (0.475)

Abbreviations: ACD, anterior chamber depth; AL, axial length; K,corneal curvature; SE, spherical equivalent.

The refractive error was measured with cycloplegic autorefraction after mydriasis (Mydrin®-P, a tropicamide compound; Santen Pharmaceutical Co., Ltd., Osaka, Japan). Ophthalmologic examinations were performed by ophthalmologists (Q.Z. and X.G.). Blood of each subject was drawn from superficial veins of the arm by using disposable syringe after sterilization of skin. Serum was removed after centrifugation of the blood and the remaining leukocytes were separated from red blood cells by hypotonic hemolysis. Leukocytes were digested by proteinase K. The digested leukocytes were then extracted by using phenol/chloroform solution. The supernatant was mixed with cold alcohol to generate a genomic DNA pellet. Genomic DNA was dissolved in TE buffer. Informed consent conforming to the tenets of the Declaration of Helsinki was obtained from each participant before the study. The Institutional Review Board of Zhongshan Ophthalmic Center approved this study.

### Mitochondrial DNA haplogroup classification

mtDNA sequence variations were scored for each sample relative to the revised Cambridge reference sequence [27]. We followed the same strategy and amplification and sequencing methods as described by Yao et al. [28], which have been used and optimized in our recent studies [10,29]. Each mtDNA was categorized according to the methods described by Yao et al. [28] and Kong [30]. Briefly, the first hypervariable segment of the mtDNA control region from 16,001 to 16,497 (HVS-I) was amplified and sequenced for each sample to allow a preliminary classification of the haplogroups. The second hypevariable segment from 30 to 407 (HVS-II) and two coding region segments (regions 2,797-3,273 and 10,171-10,659) were amplified and sequenced in certain samples to justify the haplogroup status based on the preliminary haplogroup status inferred from HVS-I. In addition, all samples were screened for the 9-bp deletion in the COII/tRNA^lys^ region by nondenaturing polyacrylamide gel (8%) electrophoresis to determine the haplogroup B status. Furthermore, haplogroups A, D, and M7 were also genotyped by restriction fragment length polymorphism (RFLP) to further confirm the inferred haplogroup status. We followed the strategy for data quality control according to the rules and guidelines described in previous reports [31,32]. This included careful handling to avoid sample contamination, double checking of the sequence reading of HVS-I and HVS-II to avoid base shift or variation missing, cautiously score transition, transversion, deletion, or insertion. For those regions genotyped by RFLP analysis, randomly selected samples were further confirmed by additional sequencing analysis. Final data of mtDNA haplogroups were independently checked by two coauthors.

### Statistical analysis

The haplogroup distribution frequencies between the two groups were analyzed by the Pearson χ^2^ test. Principal component analysis was conducted to assess the geographic origin of the study subjects based on the mtDNA haplogroup distribution frequencies. Previously reported Han Chinese mtDNA data, including those from Guangdong Province, and our previously published data were used for comparisons ([26,28] and references therein).

## Results

The mtDNA sequence variations and haplogroup classifications of all 192 subjects with and without myopia are listed in Appendix 1. All the lineages belonged to haplogroups that are found in Han Chinese and East Asian populations [28]. Most of the samples could be allocated to the smallest haplogroups, with the exception of seven samples with a status of M*, which could not be further classified based on the available information. Haplogroups D, B, F, and M7 were detected in 25 (26.04%), 19 (19.79%), 15 (15.63%), and 12 (12.50%) subjects with myopia, respectively, accounting for 73.96% of the case subjects. Similarly, these four haplogroups were present in 22 (22.92%), 18 (18.75%), 16 (16.67%), and 10 (10.42%) subjects without myopia, respectively, accounting for 68.75% of the control subjects. The haplogroup distributions between these two groups showed no statistical difference (χ^2^ test, χ^2^=11.654, p=0.556; Table 2).

**Table 2:** mtDNA haplogroup distribution frequencies (%) of subjects with and without myopia.

**Haplogroup**	**Myopias (CS1M, n=96)**	**Controls (CS1NC, n=96)**	CS1** (n=192)	**CS2# (n=102)**
B	19.79	18.75	19.3	16.7
F	15.63	16.67	16.2	19.6
M7	12.50	10.42	11.5	13.7
R†	2.08	6.25	4.2	1.9
M33	0	1.04	0.5	0.0
D	26.04	22.92	24.5	25.5
M10	3.13	2.08	2.6	2.9
M12	1.04	0	0.5	0.0
A	5.21	2.08	3.6	2.9
G	2.08	4.17	3.1	2.9
M8‡	4.17	9.38	6.8	5.9
N9a	2.08	4.17	3.1	5.9
Y	1.04	0	0.5	0.9
M*	5.21	2.08	3.6	0.9
χ^2^ test	χ^2^=11.654	p=0.556	χ^2^=6.411	p=0.930

The double asterisk indicates subjects with myopia (CS1M) and without myopia (CS1NC) in the present study. The sharp (hash mark) indicates that the Chaoshanese mtDNA data were taken from a recent report [26]. The dagger indicates that R includes R*, R9b, R9c, and R11 and the double dagger indicates that M8 includes M8a, C, and Z.

The 9-bp (CCCCCTCTA) deletion was found in sample NC960, which belongs to haplogroup F2b. The presence of this deletion in haplogroups D4b1b2 and B (including its subhaplogroups) as a haplogroup-specific variant suggests multiple origins of the 9-bp deletion [33]. We found the southern Han prevalent haplogroups (B, F, M7, and R) and northern Han prevalent haplogroups (D, G, M8a, C, and Z) in subjects with and without myopia but again with no statistical difference (χ^2^=1.377, p=0.502) in the distribution of these haplogroups between the two groups.

**Figure 1:**
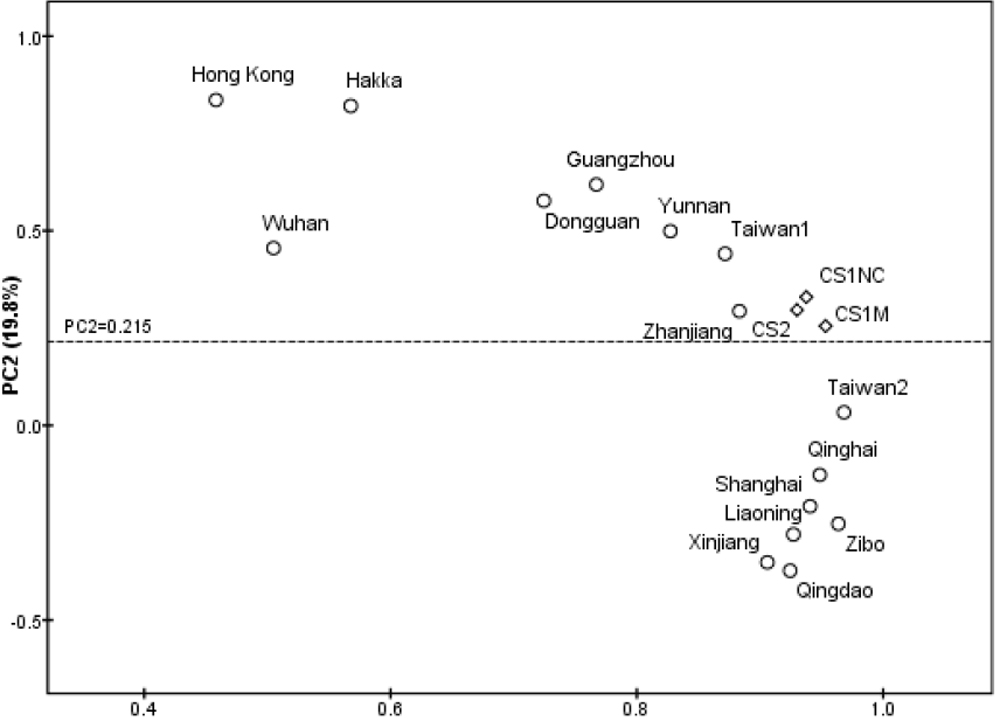
Principal component map of mitochondrial DNA variation. The mitochondrial DNA data (with respect to the haplogroup frequencies in Table 2) of 16 reported regional Han populations were from references ([26,28] and references therein). The three Chaoshanese populations are marked by diamonds, whereas other Han Chinese populations are labeled by circles with city or province names above the circle. CS1M and CS1NC represent the Chaoshanese populations with (CS1M) and without (CS1NC) myopia in this study. CS2 indicates that the Chaoshanese mtDNA data were taken from a recent report [26]. This figure demonstrated that the Chaoshanese population in this study is identical to the Chaoshanese population previously reported but is different from other Chinese population based on mitochondrial DNA haplogroup analysis.

We performed principal component analysis (Figure 1) based on the mtDNA haplogroup frequencies of the Chaoshanese populations with and without myopia and other reported Han Chinese populations ([26,28] and references therein). The first two principal components accounted for 91.8% of the genetic variation. The south-to-north cline of the populations and the heterogeneity of the southern populations were further confirmed by the second principal component (PC). The Chaoshanese population (marked as CS2 in Figure 1) reported by Wang et al. [26] showed a close affinity to the myopia and control populations (CS1M and CS1NC) in this study, which is consistent with the sampling location. This pattern suggests that the Chaoshanese are relatively homogenous as compared to other Han Chinese.

## Discussion

The mitochondrial genome encodes the oxidative phosphorylation system where energy and ROS are generated [1]. Generation of ROS can cause deleterious peroxidation of lipids, modification of proteins, and cleavage of DNA [34], which is referred to as oxidative stress. The retina is particularly sensitive to the deleterious effects of ROS because of its high oxygen consumption and its constant exposure to light [35]. Previous studies have demonstrated that exposure to oxidative stress caused degeneration of photoreceptors and other cells of the neural retina in animal models [36]. The level of lipid peroxidation products may relate to the degree of myopia [37]. Furthermore, single nucleotide polymorphisms in the mitofusin-1 (*MFN1*) and presenilin associated rhomboid-like (*PSARL*) genes are among the clustering peak showing a genetic association with myopia that was mapped to 3q26 (MYP8 locus) [15]. Both *MFN1* and *PSARL* encode mitochondrial membrane proteins that interact with Optic Atrophy 1 (OPA1), a mitochondrial protein known to cause retinal neuron degeneration when mutated [15,38]. Mitochondrial dysfunction, which is caused by mutations in either mtDNA or nuclear-encoded mitochondrial genes, can be a potential target for genetic predisposition to myopia.

In this study, we analyzed mtDNA haplogroup distributional patterns in 192 Chaoshanese individuals (including 96 with myopia and 96 without myopia) to test whether an mtDNA background would affect the clinical expression of myopia. The case and control populations presented a very similar matrilineal structure. We found no statistical difference in the frequency of certain haplogroups between the cases and controls. Principal component analysis demonstrated homogeneity of the Chaoshanese populations analyzed in this study, and this homogeneity had been previously reported [26]. It is unlikely, therefore, that an mtDNA haplogroup would affect myopia. This is in contrast to our recent observation of an increased risk of haplogroup M7b1’2 and a protective role of M8a during the expression of LHON in Chinese families with m.11778G>A [10].

To our knowledge this is the first study to examine the potential association of an mtDNA haplogroup with myopia. We failed to find any evidence that would suggest the involvement of an mtDNA background in the predisposition to myopia. Although the sample size in this study was not large, we have every reason to believe that an mtDNA background is unlikely to play a major role in myopia predisposition as our study has shown that the Chaoshanese population has high genetic homogeneity. This pattern is consistent with the relatively isolated status of the Chaoshanese. The current results may provide guidance for genome-wide association studies of myopia when selecting study populations. The case-control series from the Chaoshan area is a good candidate for such a study.

## Appendix

mtDNA sequence variation and haplogroup classification of 96 subjects with myopia (M) and 96 subjects without myopia (NC).****To access the data, click or select the words “Appendix 1.” This will initiate the download of a compressed (pdf) archive that contains the file.
